# Effects of Osseodensification on Primary Stability of Cylindrical and Conical Implants—An Ex Vivo Study

**DOI:** 10.3390/jcm12113736

**Published:** 2023-05-29

**Authors:** Márcio de Carvalho Formiga, Helio Doyle Pereira da Silva, Bruna Ghiraldini, Rafael Shinoske Siroma, Lavinia Cosmina Ardelean, Adriano Piattelli, Jamil Awad Shibli

**Affiliations:** 1 Department of Periodontology and Oral Implantology, Unisul, Palhoça, 515 Felipe Schmidt Str., Florianopolis 88101-001, SC, Brazil; marciocformiga@gmail.com; 2 Department of Periodontology and Oral Implantology, Dental Research Division, Guarulhos University, 88 Praça Tereza Cristina Sq., Guarulhos 07011-010, SC, Brazil; helio.silva@prof.ung.br (H.D.P.d.S.); rafaelshinoske@gmail.com (R.S.S.); jshibli@ung.br (J.A.S.); 3 Dental Research Division, Paulista University, 303 Borges de Figueiredo Str., São Paulo 03110-010, SP, Brazil; bruna.ghiraldini@sinimplante.com.br; 4 Department of Technology of Materials and Devices in Dental Medicine, Faculty of Dental Medicine, Multidisciplinary Center for Research, Evaluation, Diagnosis and Therapies in Oral Medicine, “Victor Babes” University of Medicine and Pharmacy Timisoara, 2 Eftimie Murgu Sq., 300041 Timisoara, Romania; 5 Department of Medical, Oral and Biotechnological Sciences, University “G. D’Annunzio” of Chieti-Pescara, 332 Viale Abruzzo Str., 66100 Chieti, Italy; apiattelli@unich.it

**Keywords:** dental implants, osseodensification, primary stability, macrogeometry

## Abstract

Primary stability is an important factor for dental implant success. In the past years, a new method for bone site preparation was introduced, named osseodensification (OD). OD produces a condensation of the trabecular portion of the bone, increasing bone-to-implant contact and primary stability. This study aims to compare the effect of OD in cylindrical and conical implants to conventional instrumentation. A total of forty implants, divided into four groups, were placed in porcine tibia: cylindrical conventional (1a), cylindrical OD (1b), conical conventional (2a) and conical OD (2b). Each implant was measured for implant stability quotient (ISQ), insertion torque (IT) and removal torque (RT). Group 2b showed the higher values for each of the evaluated parameters; groups 1b and 2b showed better results than 1a and 2a, respectively. Regarding the IT and RT, group 1b achieved higher values than group 2a, but not for ISQ. The inter-group comparison showed significant difference between groups 1a vs 2a, 1a vs 2b and 1b vs 2b for ISQ and 1a vs 1b and 1a vs 2b for RT analysis. OD resulted in improved ISQ, IT and RT of both cylindrical and conical implants.

## 1. Introduction

Dental implants represent one of the greatest advances in oral rehabilitation. Initially indicated for specific patients for lower full arch fixed rehabilitation combined with upper arch denture, it underwent many improvements which led to broadening the indications in the cases of partial, upper total fixed or removable dentures and single elements in situations where the aesthetic result is mandatory [1,2,3]. The treatment time also decreased, due to evolutions in implant macro- and microgeometry, surface treatment, types of implant/abutment connection [4,5,6,7,8]. In addition, the technique of bone instrumentation of the implant site is an important factor that has been intensively studied in order to improve the primary stability of dental implants, especially in areas with low density bone [9,10,11].

Primary stability of dental implants is an important factor to achieve clinical success, at least, in the early stages of bone healing. Some techniques are based on sub-instrumentation procedures, aiming to increase the initial bone-to-implant contact and bone density around the implant, especially in areas of type IV bone (Misch classification) [12,13,14,15]. However, the sub-instrumentation technique is not always achieved, and therefore it might compromise the secondary implant stability [10,16,17].

Osseodensification (OD) is an osteotomy bed technique that preserves the bulk bone and increases bone density by compacting the bone from the instrumentation itself, causing expansion of the ridge and increasing its density [18]. The resulting bone quality around the implants can be improved, increasing primary stability torques even in unfavorable situations. Several in vitro [18,19], animal [20,21,22,23] and human studies [24,25] have been carried out, demonstrating the improvement of the previously mentioned biological factors in the peri-implant bone, which leads to a greater probability of treatment success [26]. Moreover, the total treatment time, compared to the traditional techniques, is reduced, leading to greater patient satisfaction. OD burs can be used in both conical and cylindrical macrogeometry implants, but, as conical ones have been predominant, the majority of the clinical studies evaluated dental implants with conical macrogeometry [21,23,24,26,27].

A recent ex vivo study [28] aimed to compare OD instrumentation with under-drilling osteotomies, regarding the primary stability of the implants, using the Ostell device, and the changes in the surrounding bone density, using porcine sternums. OD bone instrumentation, showed the highest value on implant stability and increased the bone density around the implant sites, pointing out the benefits of the technique.

A human cadaver study [29] tested the temperature changes during conventional and OD instrumentation, concluding that neither of the preparation systems tested caused an increasing in temperature that would interfere negatively with the osseointegration process.

Aiming to test the healing of an implant site prepared with different osteotomy techniques, in a low-density bone ovine model, another study [30] compared conventional drilling with OD, assessing bone repair, after a 3 and 6 weeks healing period. The results indicated that the sites prepared with OD showed higher quantity of new bone formation and higher bone-to-implant contact (BIC) at 3 and 6 weeks, resulting in better osseointegration compared to conventional drilling.

Another ovine model study, that tested lumbar fixation with pedicle screws, with conventional and OD bone instrumentation [31] showed no significant differences in bone area fraction occupancy (BAFO) between the techniques. However, the mechanical tests carried out revealed that OD instrumentation provided higher degrees of implant biomechanical fixation, compared to conventional instrumentation.

Similar results were found in another ovine model study [32] that tested sub-instrumentation and OD for BIC and BAFO after 14 and 28 days. Although no significant differences on BIC and BAFO were found, OD allowed a wider implant site preparation without prejudice on primary stability and bone remodeling.

In a recent randomized clinical trial [33] comparing OD instrumentation and undersized drilling protocol, the authors achieved 100% survival rates for both groups. Interestingly, the results showed that, even with a wider implant site preparation, the OD group reached installation torques higher than the undersized drilling group.

Because of the expansion caused by bone compaction, OD may be used for crestal [34] or [24,35] of narrow ridges expansion with minimum chances to cause fenestration or dehiscence on the bone, as well as for septum expansion for immediate implant placement [36,37,38]. Another practical and very useful indication for OD is crestal sinus augmentation as an alternative to the lateral window technique [18,25,39,40], with very encouraging results because of the less traumatic, simplified and minimally invasive method of elevating the sinus membrane, which leads to less morbidity and minor surgical time.

Since most of the available studies on OD concern either conical or cylindrical implants, the aim of this ex vivo study was to compare the level of initial stability by assessing the implant stability quotient (ISQ), initial insertion torque (IT) and removal torque (RT) of dental implants with different macro geometry, cylindrical and conical, inserted in fresh porcine bone tibia, comparing the conventional bone instrumentation and OD in both macrogeometries.

## 2. Materials and Methods

### 2.1. Sample Preparation

An experimental ex vivo study model was designed, using fresh commercially available porcine tibia cuts, belonging to animals of the same age and gender, after removal of all attached soft tissue and exposing a flat surface of medullar bone, similar to bone density III-IV, as described by Misch [41]. Since no animal sacrifice was carried out, and this is not a clinical study involving humans, there was no need for an Ethical Committee approval for this study, according to Resolution from the Brazilian Health Ministry (issued on 6 May 2022, Chapter IX, art. 26, paragraph X). It was determined by ANOVA post hoc analysis that a sample size of 10 implants per group is necessary to provide a 90% power with an α of 0.05. The implants used are Tryon, 4.0 × 11.5mm (SIN, São Paulo, Brazil), double acid etched, of different macrogeometrical format, conical (Tryon Sc) and cylindrical (Tryon St).

Four different groups with 10 implant sites each were selected based on the osteotomy technique (the drilling sequences are being suggested by the manufacturers for bone instrumentation in types III and IV):

Group 1a—Conventional instrumentation for 4.0 cylindrical implants Tryon St (SIN Implants, São Paulo, Brazil) in type III-IV bone: FRLTD 2020, FHTD 2015, FPTD 2030, FHTD 3015 drills (SIN Implants, São Paulo, Brazil). All drills were used in clockwise rotation.
Group 1b—OD instrumentation (Versah, Jackson MI, USA) for 4.0 cylindrical implants Tryon St (SIN Implants, São Paulo, Brazil) in type III-IV bone: pilot (using clockwise rotation, cutting mode) and Densah VT 1828, VT 2838, VS 3238 (using counter clockwise rotation, densifying mode).
Group 2a—Conventional instrumentation for 4.0 conical implants Tryon Sc (SIN Implants, São Paulo, Brazil) in type III-IV bone: FRLTD 2020, FHTD 2015, FPTD 2030, FTCD 35, FTCD 40 drills (SIN Implants, São Paulo, Brazil). All drills were used in clockwise rotation.
Group 2b—OD instrumentation (Versah, Jackson MI, USA) for 4.0 conical implants Tryon Sc (SIN Implants, São Paulo, Brazil) in type III-IV bone: pilot (using clockwise rotation, cutting mode) and Densah VT 1828, VT 2838 (using counter clockwise rotation, densifying mode).

The usage of an extra drill in group 1b, compared to group 2b, namely VS 3238 (counter clockwise rotation), results from the necessity to properly shape the apex for inserting cylindrical implants and complies with the protocol recommended by the manufacturer. The different macrogeometry is also reflected by the drilling protocol used in groups 1a and 2a.

**Figure 1:**
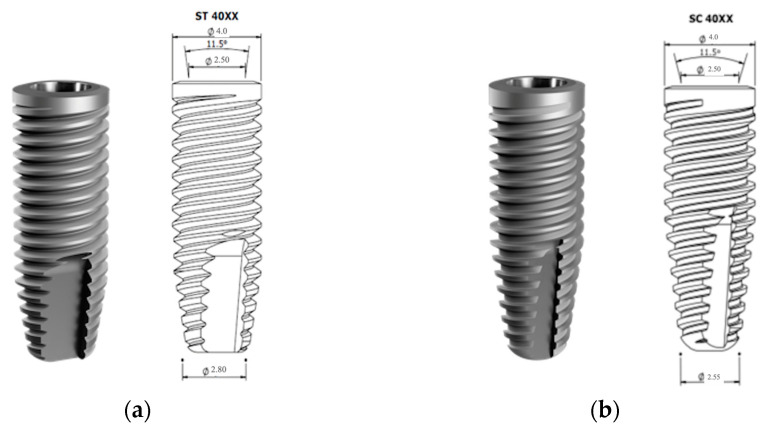
Implant characteristics and design: (**a**) Tryon St (SIN, São Paulo, Brazil); (**b**) Tryon Sc (SIN, São Paulo, Brazil).

The characteristics and design of Tryon St and Tryon Sc implants (SIN Implants, São Paulo, Brazil) are shown in Figure 1a,b.

**Figure 2:**
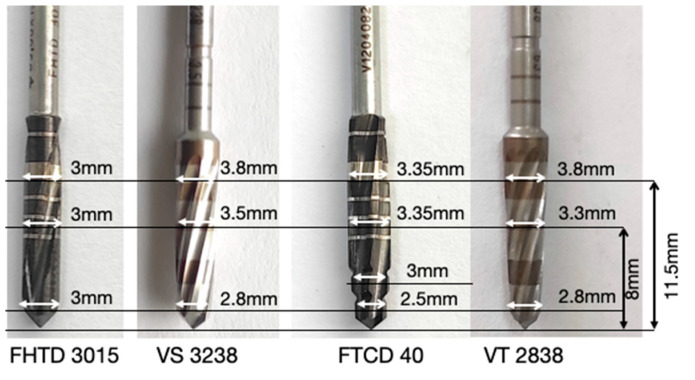
The configuration and dimensions of the final drill used for each group (1a, 1b, 2a and 2b).

Figure 2 pictures the final drill used for each group (1a, 1b, 2a and 2b), pointing out the configuration particularities of each drill.

**Figure 3:**
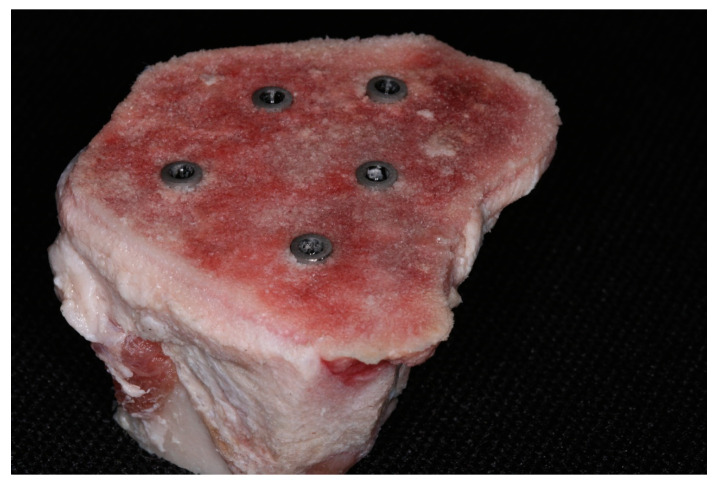
Implants placed in porcine tibia.

The implants are being characterized by the same diameter, length and surface topography, the difference consisting only in macrogeometry—cylindrical or conical. Instrumentation was performed under abundant saline irrigation, at 1200 RPM, 50N torque, by means of an NSK Surgic Pro (NSK-Nakanishi, Tochigi, Japan) surgical motor, using a NSK 20:1 handpiece (NSK-Nakanishi, Tochigi, Japan) by the same operator, in order to avoid inter-operator discrepancies. All implants were installed at bone level (Figure 3).

### 2.2. IT, ISQ and RT Assessment

**Figure 4:**
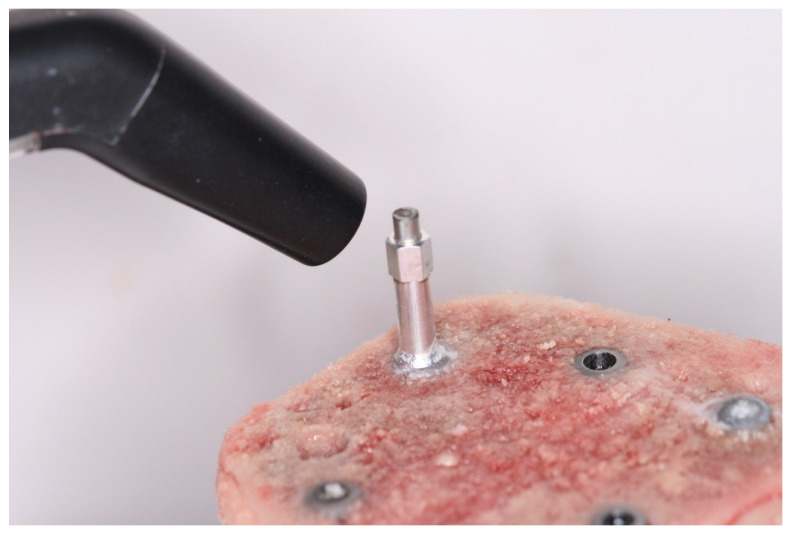
Implant stability quotient (ISQ) measurement of implant placed in the porcine tibia.

The surgical motor was used with an auto-setting of 10 RPM for implant insertion, with the torque adjusted at 10 Ncm. The value is being gradually increased by 5 Ncm after each stall of the handpiece in order to allow registering the peak value of the IT, when the implant reaches bone level position. The last torque on the motor that stalled the handpiece was recorded. The ISQ values were assessed using the Osstell Beacon (Ostell, Goteborg, Sweden), by registering the value on the four faces of each implant (anterior, posterior, medial and lateral, considering the anatomical position of the tibia) (Figure 4), and the average value was assigned for each implant [42]. The RT was noted as the last torque assigned on the motor, settled on reverse direction and starting at 5 Ncm, that would be able to move the implant in the counterclockwise direction and was increased by 5 Ncm until the implant started moving. The motor was checked and calibrated after each test to ensure the integrity of the results.

### 2.3. Statistical Analysis

The statistical analysis of the results was performed using a STATA 13 software (StataCorp LLC., College Station, TX, USA). The ANOVA test was performed to evaluate the distribution of the continuous variable (IT, ISQ, RT) between groups. A *p*-value of 0.05 was established as the level of significance. Descriptive statistics (mean, median, standard deviation, interquartile range, minimum, maximum and statistical graphs) were calculated for quantitative variables. The premises for applying variance analysis (one-way ANOVA) were checked through normality tests, variance homogeneity tests and adjustments via statistical tests (Fisher test, in the case of homogeneity and Welch test in the case of heterogeneity) as well as visualizations of the results through graphs. The Shapiro–Wilk test was used to verify the normality of the samples in each group and in each quantitative variable. Fits were observed via tests and QQ-plot graphs. Bartlett’s test was used to verify homogeneity of variances. In the case of statistical significance, in the presence of homogeneous variances, Tukey post hoc tests were performed, and in the presence of nonhomogeneous variance, Games–Howell tests were used. For the entire analysis, a statistical significance level of 5% was considered. The free Jamovi [43], R [44] and Python [45,46] software was used to create tables, graphs and statistical analysis.

## 3. Results

A total of 40 implant site instrumentations (*n* = 10 for each group) were assessed in this study, divided in four groups (1a, 1b, 2a and 2b). All bone instrumentations and implant placements were performed by the same experienced operator.

ISQ value ranged between 44 for group 1a to 61.5 for group 2b. Mean values of ISQ were 48.6 ± 3.3, 50.8 ± 3.0, 53.2 ± 2.6 and 55.3 ± 3.1 for groups 1a, 1b, 2a and 2b, respectively. Inter-group analysis showed a significant difference between groups 1a vs 2a, 1a vs 2b and 1b vs 2b. OD instrumentation groups (1b, 2b) reached higher ISQ values than those of conventional instrumentation groups (1a, 2a), and conical implants (group 2a, 2b) reached higher ISQ values than those of cylindrical implants (group 1a, 1b).

Regarding IT, its minimum values are quite similar for all groups, and its maximum values vary from 30 Ncm (group 1a) to 60 Ncm (group 2b). No significant difference was noted (*p* > 0.05). RT showed a significant difference between groups, with the lowest mean RT of 8.5 ± 4.7 for group 1a and the highest mean RT of 30.0 ± 18.1 for group 2b (Table 1 and Table 2).

The IT and RT showed a similar behavior, with group 1b reaching higher values than group 2a, which was not the case of the ISQ measurements.

The measured values for all the samples and parameters are given in Appendix A.

## 4. Discussion

The results of this study highlight that OD bone instrumentation for conical implants (group 2b) showed higher values for all analyzed variables. The second place for ISQ measurements belongs to the conventional bone instrumentation for conical implants (group 2a), while for IT and RT, OD bone instrumentation for cylindrical implants (group 1b) showed higher values. When comparing the techniques, OD showed improved parameters for both conical and cylindrical implant macrogeometries. These results are in accordance with previous published in vitro and ex vivo animal studies [18,19,28,29,30,31,32,42,47] which aimed to compare initial implant stability with/without OD bone instrumentation.

Conical implants are known to achieve better primary stability than cylindrical implants [48,49], which is an important factor for implant success [50,51,52], in accordance with our findings when comparing group 1a with 2a or 1b with 2b. The difference in results related to implant macrogeometry was evident in this study, either for conventional or OD instrumentation. When comparing group 1a with 2a and 1b with 2b, significant differences in the ISQ values are to be found. Implants from the same manufacturer, with the same surface treatment, threads, diameter and length, were compared in our study. The only difference was in their geometry, resulting in diminished bias and highlighting the better primary stability of conical implants over cylindrical ones [48,49].

Regarding IT and RT, the results of this study showed that the OD technique aided the cylindrical group (1b) to achieve higher values than the conical conventional one (2a). Similar results were found in animal studies [20,22,23], reporting that OD resulted in a higher resistance for implant removal and higher IT values compared to conventional drilling. Our findings are also in accordance with recent studies which showed that OD could improve the bone-to-implant contact by dynamic condensation of the bone [53,54]. Both parameters had the same pattern in the results of all groups. Analyzing the results (Appendix A) for RT, both OD groups (1b, 2b) achieved values closer to the IT ones, in comparison with the conventional instrumentation groups (1a, 2a), and, in some cases, even higher values were reported. This fact may be explained by the “spring-back effect” caused by the OD bone instrumentation burs used in our study and may also be the reason why previous published clinical studies showed higher IT, when alveolar ridge was instrumented by OD with the final bur close to the implant diameter, and yet with higher values when compared to sub-instrumentation, carried out with a thinner final bur [18]. This occurred when using both cylindrical and conical implants and may be the reason why ISQ and RT resulted in significant differences between the groups, but not the IT.

Undersized instrumentation is a valid way to increase primary stability [12,13,14], this being the protocol indicated by the implant manufacturer (SIN, São Paulo, Brazil), and used in this study, for low density bone porcine model. The OD protocol, however, suggests that the last drill should be a little closer to the diameter of the implant to be placed [18]. In our study, the OD protocol was performed, with the last drill diameter being closer to the implant size and wider than the one used for the conventional protocol. This is in accordance with clinical studies that compared undersized drilling osteotomies with OD and concluded that OD groups reached higher IT than undersized drilling [28,33,34]. Nevertheless, both OD groups in our study performed better than the conventional similar groups. The higher ISQ displayed by the conical conventional instrumentation (group 2a) compared to the cylindrical OD one (group 1b) is probably due to the undersized drilling of the first one, also because the design of the drill was made to fit with the implant design [55], but this improvement did not reflect in the IT and RT analysis.

The reason of using a porcine tibia [18,19,28,42,47] for this study, is because it consists mostly of type III and IV bone density, being a highly cancellous bone, that mimics the lower bone density areas of human jaws (upper posterior region), and this requires an increased primary stability of implants. Furthermore, it has been established that, in order to be condensed, the region of bone instrumentation must have less cortical and more medullar structure [17,18,19,20,21]. By increasing the ratio of bone-to-implant contact, primary stability increases and consequently the ability of the implant to withstand micromovements, which could lead to flaws in the osseointegration process [56,57]. By avoiding this problem, the transition from primary to secondary stability may occur by bone remodeling around a dental implant [20]. Because even the micro and nanogeometry of the implant surface may interfere with the process, our decision was to use implants with the same surface treatment, and even the same thread pattern [58,59], to eliminate as much bias as possible in this study. Other studies have compared results on primary stability of implants placed on synthetic polyurethane blocks [55,56,57,58,59,60,61,62,63]. However, if we consider OD, these materials are not the best choice for a real evaluation, as there is no collagen in the synthetic blocks, and it has been established that this is crucial for the technique [18]. Therefore, studies of OD carried out on synthetic blocks usually do not show the same results as ex vivo animal [18,19,28,29,42,47,64], in vivo animal [20,21,22,23,30,31,32,33] or clinical studies [24,25,26,34,35,36,37,38,39,40,53,54].

A recent systematic review that aimed to evaluate the instruments used for implant site preparation [65] compared conventional drills, osteotomes, a piezoelectric device, Er:YAG Laser and OD burs. Although it was concluded that OD did not improve BIC comparing with conventional drilling, it was mentioned that OD showed promising results because of the significant increase in the biomechanical properties [31]. However, it is worth mentioning that in 2018, the year of the publication, there were not many OD clinical studies available.

Most recently, a systematic review about OD [66], highlighted its advantages over conventional drilling, regarding BIC and BAFO. It also pointed higher IT values for OD. As most of the studies included in this systematic review were nonclinical, there should be caution before extrapolating the results to clinical practice. Even so, our IT results were similar.

Another recently published systematic review [67], aiming to compare implant stability of OD to conventional drilling, included only clinical studies. With three studies selected for the analysis, results showed that OD presented consistently higher ISQ at baseline and at 4 and 6 months after implant placement, compared to conventional drilling. Although our study was static, with only one measurement, the results are comparable with the baseline results of this systematic review, with higher ISQ for OD groups.

A review article on biomechanical considerations in implant dentistry [8] also highlighted the effectiveness of OD in improving biomechanical properties and thus secondary stability of implants in translational studies, with advantages over conventional drilling techniques, but suggested that more research is necessary to identify the ideal characteristics of instrumentation protocols, implants’ macro and microgeometries, as well as host characteristics, to optimize osseointegration.

The limitation of this study is due to the method used for the IT and RT measurement, which does not deliver an exact numerical value, but a closer one, jumping from each 5 Ncm. However, this methodology proved to be easily accessible for clinicians and surgeons in daily implant practice. Another point is that ISQ is a valid parameter to assess the mechanical stability of the implant over time and is considered to have predictive power for the clinical outcome [68], but may be questionable in static studies [69]. More clinical long-term studies are necessary to confirm the effects of OD on osseointegration.

## 5. Conclusions

Considering the limitations of this study it can be concluded that OD bone instrumentation increased the level of ISQ, IT and RT, compared to conventional bone instrumentation, in bone type III and IV. It can also be concluded that cylindrical implants inserted with OD display improvements when compared to conical implants inserted with conventional instrumentation.

## Figures and Tables

**Table 1:** Comparison of mean and median ISQ, IT and RT with minimum and maximum values for each group.

Parameter	Group		Mean ± SD	Median ± IQR	Minimum	Maximum	ANOVA
							*p*-Value
ISQ	1a	Cylindrical conventional	48.6 ± 3.3	49.5 ± 4.4	44.0	54.0	0.0001
	1b	Cylindrical OD	50.8 ± 3.0	50.8 ± 2.5	45.5	55.5	
	2a	Conical conventional	53.2 ± 2.6	53.8 ± 4.6	50.0	56.5	
	2b	Conical OD	55.3 ± 3.1	54.5 ± 3.5	51.5	61.5	
IT	1a	Cylindrical conventional	16.5 ± 5.8	15.0 ± 3.8	10.0	30.0	0.0619
	1b	Cylindrical OD	26.0 ± 11.3	27.5 ± 13.8	10.0	45.0	
	2a	Conical conventional	24.5 ± 12.4	25.0 ± 15.0	10.0	45.0	
	2b	Conical OD	30.5 ± 14.0	27.5 ± 17.5	15.0	60.0	
RT	1a	Cylindrical conventional	8.5 ± 4.7	7.5 ± 5.0	5.0	20.0	0.0017
	1b	Cylindrical OD	21.5 ± 12.0	22.5 ± 13.8	5.0	40.0	
	2a	Conical conventional	18.5 ± 11.6	17.5 ± 15.0	5.0	40.0	
	2b	Conical OD	30.0 ± 18.1	22.5 ± 26.3	10.0	65.0	

ISQ: implant stability quotient, IT: insertion torque, RT: removal torque, OD: osseodensification, SD: standard deviation, IQR: inter quartile range.

**Table 2:** Inter-group comparison of mean ISQ, IT, and RT (one-way ANOVA).

Comparison	ISQ *		IT *		RT **	
	Mean Diff.	*p*_Value	Mean Diff.	*p*_Value	Mean Diff.	*p*_Value
1a vs 2a	−4.6	0.008	−8.0	0.400	−10.0	0.105
1a vs 1b	−2.3	0.346	−9.5	0.254	−13.0	0.035
1a vs 2b	−6.8	0.000	−14.0	0.062	−21.5	0.019
2a vs 1b	−2.4	0.309	1.5	0.991	3.0	0.940
2a vs 2b	−2.2	0.378	−6.0	0.638	−11.5	0.360
1b vs 2b	−4.5	0.009	−4.5	0.809	−8.5	0.614

* Tukey post hoc test. ** Games–Howell post hoc test. ISQ: implant stability quotient, IT: insertion torque, RT: removal torque

## Data Availability

The datasets used and/or analyzed during the current study are available in Appendix A.

## Appendix A

**Table A1:** Values of ISQ, IT and RT for conventional cylindrical instrumentation (group 1a).

Cylindrical Conventional Group 1a (Sample Number)	ISQ (Mean for Each Implant)	IT (Ncm)	RT (Ncm)
1	49.5	20	10
2	51.5	15	10
3	54	30	20
4	50.5	15	5
5	46	10	5
6	49.5	20	10
7	44	10	5
8	44.5	15	5
9	46	15	5
10	50	15	10

ISQ: implant stability quotient, IT: insertion torque, RT: removal torque.

**Table A2:** Values of ISQ, IT and RT for OD cylindrical instrumentation (group 1b).

Cylindrical OD Group 1b (Sample Number)	ISQ (Mean Value for Each Implant)	IT (Ncm)	RT (Ncm)
1	45.5	15	10
2	55	40	40
3	55.5	45	40
4	50	25	25
5	51	10	5
6	50.5	30	25
7	52	30	20
8	48.5	20	15
9	49	15	10
10	51	30	25

ISQ: implant stability quotient, IT: insertion torque, RT: removal torque; OD: Osseodensification.

**Table A3:** Values of ISQ, IT and RT for conventional conical instrumentation (group 2a).

Conical Conventional Group 2a (Sample Number)	ISQ (Mean Value for Each Implant)	IT (Ncm)	RT (Ncm)
1	50	15	10
2	50	20	15
3	51	10	5
4	56.5	15	10
5	55.5	30	25
6	56	30	20
7	50.5	10	5
8	54.5	45	40
9	54.5	40	30
10	53	30	25

ISQ: implant stability quotient, IT: insertion torque, RT: removal torque.

**Table A4:** Values of ISQ, IT and RT for OD conical instrumentation (group 2b).

Conical OD Group 2b (Sample Number)	ISQ (Mean Value for Each Implant)	IT (Ncm)	RT (Ncm)
1	53.5	20	15
2	54.5	15	10
3	57.5	30	25
4	52	20	20
5	54	25	20
6	54.5	30	35
7	51.5	20	15
8	55.5	40	45
9	58.5	45	50
10	61.5	60	65

ISQ: implant stability quotient, IT: insertion torque, RT: removal torque; OD: osseodensification.
